# Untargeted Metabolomics and Polyamine Profiling in Serum before and after Surgery in Colorectal Cancer Patients

**DOI:** 10.3390/metabo10120487

**Published:** 2020-11-27

**Authors:** Yu Ra Lee, Ki-Yong An, Justin Jeon, Nam Kyu Kim, Ji Won Lee, Jongki Hong, Bong Chul Chung

**Affiliations:** 1 Molecular Recognition Research Center, Korea Institute of Science and Technology, Seoul 02792, Korea; T16627@kist.re.kr; 2 KHU-KIST Department of Converging Science and Technology, Kyung Hee University, Seoul 02447, Korea; 3 Faculty of Kinesiology, Sport and Recreation, University of Alberta, Edmonton, AB T6G 2R3, Canada; kiyong1@ualberta.ca; 4 Department of Sport Industry, Yonsei University, Seoul 03722, Korea; JJEON@YONSEI.AC.KR; 5 Exercise Medicine Center for Diabetes and Cancer Patients, ICONS, Yonsei University, Seoul 03722, Korea; 6 Department of Surgery, Yonsei University College of Medicine, Seoul 03722, Korea; NAMKYUK@yuhs.ac; 7 Department of Family Medicine, Yonsei University College of Medicine, Seoul 06273, Korea; indi5645@yuhs.ac; 8 College of Pharmacy, Kyung Hee University, Seoul 02447, Korea

**Keywords:** colorectal cancer, liquid chromatography-mass spectrometry, polyamine, tumorectomy, metabolomics, biomarker

## Abstract

Colorectal cancer is one of the most prevalent cancers in Korea and globally. In this study, we aimed to characterize the differential serum metabolomic profiles between pre-operative and post-operative patients with colorectal cancer. To investigate the significant metabolites and metabolic pathways associated with colorectal cancer, we analyzed serum samples from 68 patients (aged 20–71, mean 57.57 years). Untargeted and targeted metabolomics profiling in patients with colorectal cancer were performed using liquid chromatography-mass spectrometry. Untargeted analysis identified differences in sphingolipid metabolism, steroid biosynthesis, and arginine and proline metabolism in pre- and post-operative patients with colorectal cancer. We then performed quantitative target profiling of polyamines, synthesized from arginine and proline metabolism, to identify potential polyamines that may serve as effective biomarkers for colorectal cancer. Results indicate a significantly reduced serum concentration of putrescine in post-operative patients compared to pre-operative patients. Our metabolomics approach provided insights into the physiological alterations in patients with colorectal cancer after surgery.

## 1. Introduction

Colorectal cancer (CRC) occurs in the colon, rectum, and appendix. This cancer has one of the highest global incidences, and accounts for the third most common cancer in Korea [1]. Despite an increasing understanding of the molecular etiology of CRC over the past 20 years, there remains a lack of reliable and robust biomarkers for monitoring the treatment efficacy associated with this disease. Currently, surgery, involving removal of the tumor, is the most common treatment for CRC. CRC surgery involves the removal of sufficient adjacent large intestine, including tumors, to prevent the cancer from affecting the remaining areas and to remove nearby lymph nodes and blood vessels, which provide avenues for metastases. As surgical techniques and tools have developed, the use of laparoscopy [2] and robots [3] has been applied in several cases. Using these methods, the patient experiences less stress, the incisions are small, and the patient recovers more quickly after surgery, compared to standard open surgeries.

Additionally, metabolomics provide comprehensive analysis of cellular metabolites and metabolic pathways. Specifically, it can be used to identify the levels of metabolites and their associations with metabolic processes of various pathways. Hence, it is an important research field for the identification of various disease-related biomarkers, including those of several cancers [4]. It is also crucial for the subsequent development of diagnostic methods by analyzing metabolic pathways *in vivo* to elucidate disease etiology. Specifically, untargeted profiling is used to identify the affected metabolic pathways for diagnosis and prognosis, as well as to identify potential biomarkers. Indeed, several metabolomic studies have compared the profiles of normal controls with those of patients with CRC, using urine [5] and serum [6] samples. However, no studies yet performed metabolome analysis of CRC patients before and after surgery.

Polyamines are well-known metabolite class that are correlated with cell proliferation and differentiation [7]. Moreover, polyamines have been found to be positively correlated with the abundance of cancer cells in cancer tissue and, therefore, may serve as a suitable marker for detecting the progression of various cancers [8,9]. Specifically, the polyamine spermine serves as a significant prognostic factor for CRC recurrence [10]. Meanwhile, serum and urine polyamine concentrations are associated with CRC stage; the concentration of putrescine gradually increases as tumor size increases [11]. Applying this concept, we previously performed polyamine analysis with pre- and post-operative breast cancer serum samples [12]. Using fluorescence detection, polyamine concentrations in serum and urine were found to be normalized in patients after surgery [11]. Therefore, we analyzed serum polyamine concentrations for targeted profiling using mass spectrometry before and after surgery. Meanwhile, in the current study, we performed untargeted metabolomics to identify discursive differences in the serum metabolite profiles between pre- and post-operative patients with CRC. We hypothesized that arginine and proline (the precursors of polyamines) metabolism differs after surgery and that these compounds can serve as biomarkers of cancer. Furthermore, using targeted profiling, we investigated the biological roles of polyamines in CRC.

## 2. Results

### 2.1. Metabolic Patterns Detected in Serum Samples of CRC Patients Using Untargeted Metabolomics

**Figure 1:**
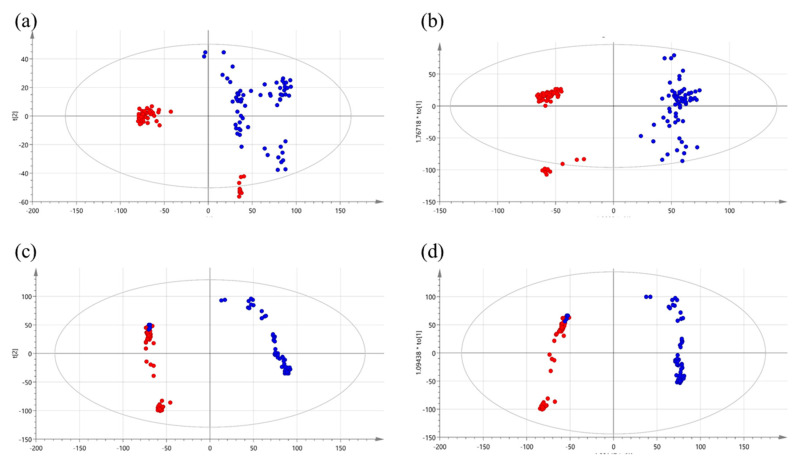
Score plots obtained using partial least squares discriminant analysis (PLS-DA) and orthogonal partial least squares discriminant analysis (OPLS-DA) for pre-operative (red circles) and post-operative (blue circles) patients with colorectal cancer. (**a**) PLS-DA in positive mode, (**b**) OPLS-DA in positive mode, (**c**) PLS-DA in negative mode, and (**d**) OPLS-DA in negative mode. PLS-DA, partial least squares discriminant analysis; OPLS-DA, orthogonal partial least squares discriminant analysis.

For discriminative pattern analysis before and after surgery, we performed partial least squares discriminant analysis (PLS-DA) and orthogonal partial least squares discriminant analysis (OPLS-DA). Analysis of the OPLS-DA score plots clearly distinguished pre- and post-operative patients. The PLS-DA score plot of the positive ionization mode data showed an accuracy of 99.3% and cross validation (R2) of 96.7%. The OPLS-DA score plot displayed clear segmentation between the two groups with a cumulative R2X of 0.274, R2Y of 0.762, and Q2 of 0.758 (Figure 1a,b). Based on the variable importance in projection (VIP) and *p*-values of the metabolites identified via the PLS-DA model (VIP > 1 and *p* < 0.05), biomarkers capable of distinguishing between pre- and post-operative CRC patients group were selected (Table 1). To confirm the regulation of each metabolite, we indicated the Pearson correlation coefficient (p(corr)) and fold change (FC) values. The FC value represented the difference between the two groups and was calculated as the average of each individual peak area: (Mean value of peak area obtained from post-surgery patients)/(mean value of peak area obtained from pre-surgery patients). If the FC value was greater than 1, the metabolites were upregulated within the post-surgery group. In addition, we performed Pearson correlation coefficient analysis to assess whether the relationship between the two groups was linear or nonlinear. Notably, 0 < ρ ≤ +1 represents a positive correlation, while −1 ≤ ρ < 0 represents a negative correlation.

Using the negative ionization mode, the PLS-DA score plot data showed an accuracy of 97.3% and cross validation (R2) of 93.5%. The OPLS-DA score plot displayed clear segmentation into the two groups with a cumulative R2X of 0.154, R2Y of 0.85, and Q2 of 0.842 (Figure 1c,d).

Based on the PLS-DA model, metabolite screening was performed. Higher VIP values had a greater impact on the discrimination of the model. A total of 22 variables met these criteria (positive ionization mode: 20 of 381 variable metabolites; negative ionization mode: 2 of 67 variable metabolites), with VIP > 1 and *p*-value < 0.05. We corrected all missing metabolite entries, total 773 values, with a low constant value, such as zero.

**Figure 2:**
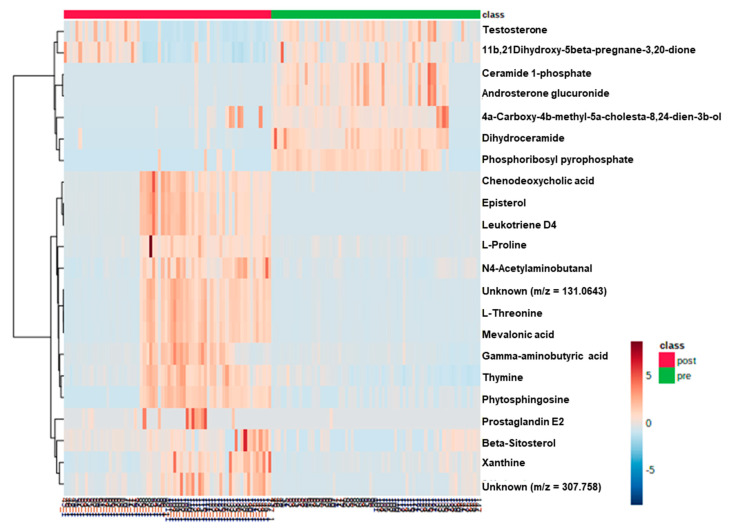
Heatmap of the 22 differentially accumulated metabolites. The heatmap provides a visualization of the changes in the abundance of metabolites specified in each row (normalized by log 10 scale). The color ranges from deep orange, indicating high abundance, to deep blue, indicating low abundance. On the top of the heatmap, green bars and red bars indicate pre-operative and post-operative patients with colorectal cancer, respectively.

Differences between the samples from pre- and post-operative CRC patients were also confirmed by comparing heatmaps performed using MetaboAnalyst 3.0. software with 22 metabolites (Figure 2). On the basis of the heatmap, the distribution of metabolites could be visually divided into those that were upregulated or downregulated.

### 2.2. Metabolic Pathway Analysis

**Figure 3:**
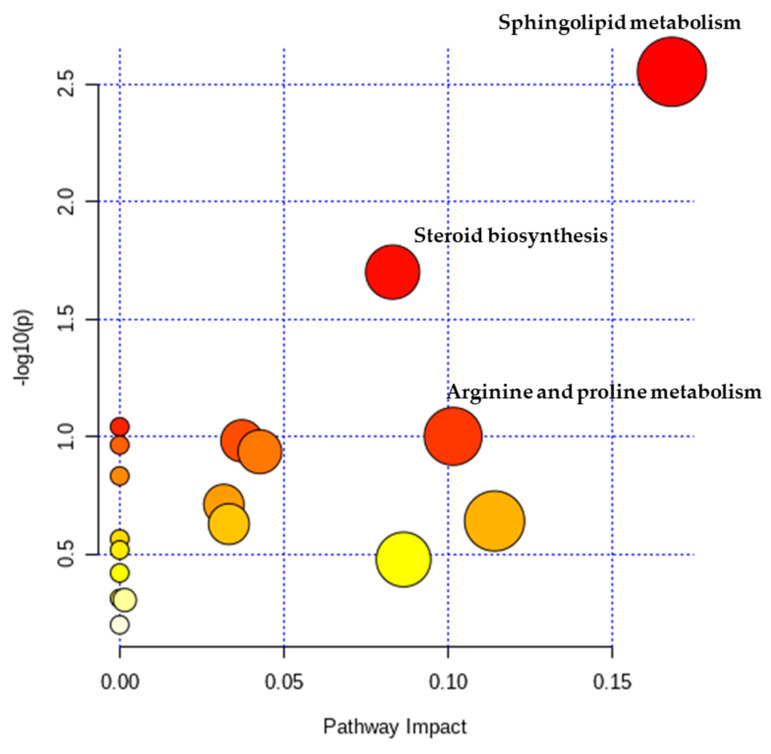
A systemic view of the disordered metabolic pathways associated with surgery. The colors (varying from yellow to red) indicate that the metabolic pathways exhibit different levels of significance, red indicating a more significant pathway than those indicated by yellow circles. All metabolic pathways have been described according to *p*-values from the pathway enrichment analysis (*y*-axis) and impact values based on the pathway topology analysis (*x*-axis).

Among all detected metabolites, 22 variables met criteria (VIP values > 1 and *p*-values < 0.05) were performed searching pathway analysis using the MetaboAnalyst 3.0. Functional pathway analysis was performed, to identify altered metabolic pathways between pre- and post-operative patients (Figure 3).

### 2.3. Quantification of Polyamines in Serum Samples of Patients with Colorectal Cancer

Using 200 μL aliquots of the serum samples, we detected nine polyamines whose concentrations varied from 2.29 to 3764.49 ng/mL (Table 2). We then quantified the metabolic profiles of serum polyamines from all pre-operative (*n* = 68) and post-operative (*n* = 68) patients with CRC.

**Figure 4:**
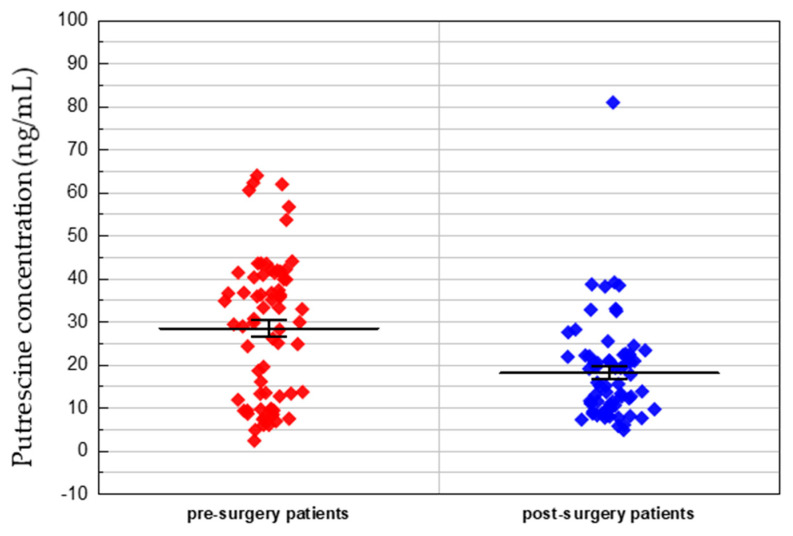
Putrescine concentration in pre- and post-operative patients with colorectal cancer. Each data point represents a patient’s individual putrescine concentration.

The Student’s t-test showed that the concentration of putrescine (PUT) was significantly decreased after surgery (pre-operative patients: Mean 28.45 ng/mL, range 2.29–63.93 ng/mL; post-operative patients: Mean 18.18 ng/mL, range 4.76–80.91 ng/mL; *p*-value, 4.97 × 10^−5^). The differences in putrescine concentrations between pre- and post-operative patients with colorectal cancer are presented in Figure 4.

## 3. Discussion

Untargeted metabolomics was performed using ultra-performance liquid chromatography-mass spectrometry (UPLC-MS). Results indicated that multiple metabolic pathways were altered between pre- and post-operative patients with CRC. The most altered pathways were those associated with sphingolipid metabolism, steroid biosynthesis, and arginine and proline metabolism.

The most significantly altered profile was that of sphingolipid metabolism. Sphingolipids are effective at inhibiting tumor formation [13]. The intracellular function of sphingolipids is diverse and related to cell growth, cell cycling, and apoptosis [14]. Ceramide, a sphingolipid metabolite, may activate cell signaling by forming a cell membrane-specific domain. Ceramide is also an important factor in apoptosis with its mechanism having been studied in depth [15]. In addition, because of an *in vitro* experiment assessing glioma treatment confirmed that apoptosis occurred when sphingosine kinase was inhibited in IDH cells and when sphingosine-1-phosphate production was inhibited [16]. However, no studies have yet been performed examining the association between phytosphingosines and CRC; meanwhile, they have been shown to induce inhibition of epithelial mesenchymal transition-related protein expression in malignant breast cancer [17]. As the number of normal cells increases after surgery, several metabolites, such as phytosphingosine, may also increase their abundance to suppress metastatic tumors. Furthermore, CRC tissues reportedly have increased expression of ceramidase synthase mRNA [18]. However, removal of tumor may alter ceramide levels. We found that the levels of compounds associated with phytosphingosine increased following surgery; however, levels of dihydroceramide and ceramide 1-phosphate decreased. As quantitative analysis of several metabolites belonging to the sphingolipid pathway does not exist, more detailed analysis of this pathway is warranted.

We also observed that steroid biosynthesis was altered following surgery. Patients with CRC excrete higher amounts of major bile acids (lithocholic acid and deoxycholic acid) compared to healthy individuals [19]. Moreover, the presence of estrogen and progesterone receptors influence the growth of colorectal tumors [20]. Among the several compounds identified, testosterone; 11β, 21-dihydroxy-5β-pregnane-3,20-dione; 4α-carboxy-4β-methyl-5α-cholesta-8,24-dien-3β-ol; and androsterone glucuronide were particularly downregulated following surgery; however, episterol and beta-sitosterol were upregulated following surgery. Reportedly, the male sex hormone, testosterone, can stimulate the growth of colon cancer [21], which is consistent with our results of downregulated testosterone levels post-surgery. Additionally, episterol is converted to beta-sitosterol via methylation by S-adenosyl methionine. Beta-sitosterol is involved in cell membrane stabilization and elicits anticancer activity [22]. However, studies on other compounds associated with steroid biosynthesis identified in our analysis have not been previously conducted in the context of CRC. In the future, it may be possible to confirm the detailed metabolic pathways using quantitative analysis of the metabolites in the steroid synthesis pathway, before and after CRC surgery.

Arginine and proline metabolism were also observed to differ before and after surgery. Gamma-aminobutyric acid and L-proline were the most significantly altered metabolites among the groups, both of which were increased after surgery. Proline oxidase is involved in p53-induced apoptosis in CRC cells [23]. In addition, when various other amino acids, such as lysine, proline, and arginine are administered to mice with colorectal cancer cells, tumor growth is strongly inhibited without any side effects [24]. Moreover, proline plays an important role in cancer metabolism. The apoptosis mechanism activated by proline oxidase is mediated by production of reactive oxygen species. In human tumors, the effect of proline oxidase is downregulated [25]. Therefore, it is important to quantify various amino acids, particularly proline, to investigate the effects of tumor resection.

After analysis of the untargeted metabolomics, we performed quantitative analysis of polyamines, which is widely known as a biomarker in cancer patients and colorectal cancer recurrence [10]. Polyamine biosynthesis was the third most altered pathway, along with arginine and proline metabolism. Therefore, we sought to determine whether the abundance of various polyamines was altered following surgery. Nine polyamines were quantitated using liquid chromatography-mass spectrometry, out of which, PUT was significantly decreased after surgery. PUT is one of the main polyamines present in humans and is produced by the conversion of ornithine; it is correlated with cell proliferation. In fact, difluoromethylornithine has been used as a chemopreventive agent for treating prostate cancer, and decreases PUT levels [26]. Additionally, macrophage-derived PUT can possibly improve the sensitivity of chemotherapy in patients with CRC [27]. In addition, PUT levels are proportional to the size of malignant tumors related to the central nervous system, such as brain tumor [28]. It has been suggested that the bioavailability of PUT has a potential effect on colon tumorigenesis and tissue regeneration in rats [29]. Moreover, increased levels of intracellular PUT have proven effective in promoting CT-26 colon tumor cell growth [30]. In vitro studies have further demonstrated that increased concentrations of PUT in culture media enhance the proliferation of colon cancer cells [30]. This can be interpreted indirectly as follows: Surgical removal of the CRC tumor would result in a decrease in the PUT concentration, which is consistent with our current findings. Although many studies have reported elevated levels of polyamines in cancer patients, few have examined the effect on polyamine concentration following surgery, therefore a detailed study on the quantitative analysis of polyamine levels, particularly PUT levels, before and after surgery is needed. Hence, polyamine analysis may enable biochemical monitoring of patients before and after surgery and may be effective for studying other types of tumors.

## 4. Materials and Methods

### 4.1. Chemicals and Materials

All chemicals used for this experiment were obtained from Sigma-Aldrich (St. Louis, MO, USA). All solvents used were purchased from Burdick & Jackson (Muskegon, MI, USA). Water was filtered using a Millipore Milli-Q purification system (Bedford, MA, USA).

### 4.2. Serum Sample Collection

Sixty-eight patients with CRC (49 male and 19 female) over 20 years of age, were recruited for this study from Yonsei University College of Medicine, as summarized in Table 3. Exclusion criteria applied for this study include patients who were previously treated with chemotherapy or radiation. All blood samples were collected in the morning after a 12-h fast. Samples were collected immediately just before surgery and the day after surgery from each eligible patient. On the day of surgery, anesthesia was administered intravenously using fentanyl and ramosetron to ensure a constant dose for 24–30 h. The study was approved by the Institutional Review Board of Severance Hospital (IRB No. 4-2010-0147). Informed consent was obtained from each participant before collecting samples. Whole blood was centrifuged at 3000 rpm for 15 min to obtain the serum, which was then stored at −80 °C until analysis. The tumor classification, histological grade, and lymph node metastasis status of individual tumor samples were evaluated by pathologists blinded to the samples, according to the tumor-node-metastasis classification of the International Union against Cancer (Edition 7).

### 4.3. Sample Preparation for Untargeted Metabolic Profiling

Serum samples for the untargeted metabolic profiling were prepared in 100 μL aliquots, and 400 μL of acetonitrile was added for protein precipitation, followed by centrifugation with Ultrafree^®^-MC-VV centrifugal filters (MilliporeSigma, Burlington, MA, USA) at 1200× *g* for 5 min.

### 4.4. UPLC-MS

The spectrometry conditions applied were the same as those described previously [31]. Metabolic profiling was performed using an ACQUITY™ ultra-performance liquid chromatography system (Waters, Milford, MA, USA) coupled to a Q-Tof Premier™ quadrupole/time-of-flight hybrid mass spectrometer system from Waters (Milford, MA, USA). Chromatographic separation was conducted using an ACQUITY UPLC BEH C18 (2.1 × 100 mm, 1.7 μm) column (Waters, Milford, MA, USA) at 0.35 mL/min. The gradient elution system consisted of solvent A (water with 0.1% formic acid) and solvent B (acetonitrile with 0.1% formic acid) and was controlled as follows: 0–3 min, 5% B; 3–10 min, 5–50% B; 10–11.5 min, 50–95% B; 11.5–12 min, 95–5% B. The gradient was then returned to the initial concentration (5% B) and held for 2 min before running the next sample. The column and autosampler temperatures were maintained at 40 and 4 °C, respectively. The sample injection volume was 5 μL. Subsequently, mass spectrometer was operated in both positive and negative ionization modes for precise mass measurements. The sample was analyzed under the full scan mode, and the m/z range was set to 50–1200 with a mass window of 0.05 Da. The data were processed using the MassLynx 4.1 software (Milford, MA, USA).

### 4.5. Sample Preparation for Targeted Profiling

For targeted profiling, we added 800 µL of acetonitrile for protein precipitation to 200 µL of each plasma sample, followed by the addition of an internal standard, 1,6-diaminohexane (1 ppm × 20 µL). After protein precipitation, samples were centrifuged at 1200× *g* for 5 min with Smart R17 plus (Hanil, Kimpo, Korea). The supernatants were transferred to a 10-mL tube to which 100 µL of dansyl chloride (4 mg/mL in acetonitrile) and 100 µL of sodium carbonate buffer (0.1 M, pH 9.0) were added. The mixture was incubated at 60 °C for 15 min. After evaporation, the residue was reconstituted with 100 µL of methanol.

### 4.6. LC-MS Profiling of Polyamines

Chromatography was performed using a Shiseido nanospace SI-2 HPLC system (Osaka Soda, Osaka, Japan) coupled to an LTQ XL ion trap MS (Thermo Fisher Scientific, Waltham, MA, USA). Solvent A comprised water with 0.1% formic acid in 5% acetonitrile, and solvent B comprised acetonitrile with 0.1% formic acid in 5% water. These were used to run samples on a Hypersil GOLD C18 column (150 × 2.1 mm inner diameter; 3 µm; Thermo Fisher, Waltham, MA, USA) under the following gradient conditions: 0 min, 12% B; 0–17 min, 12 to 88% B (hold for 8 min); 25–28 min, 88 to 12% B, using a flow rate of 0.2 mL/min at 35 °C. The sample injection volume was 5 µL. Mass spectrometer was operated in positive mode with electrospray ionization. Subsequently, raw data were collected and processed using the X-Calibur software (Thermo Fisher Scientific, Waltham, MA, USA).

### 4.7. Statistical Analysis

Using MassLynx 4.1 software, we evaluated the value of retention time and mass/charge (m/z). MassLynx software was used to process raw data with baseline correction, scaling, peak alignment, and matrix manipulation. The MarkerLynx application manager for MassLynx software accurately detects ions of interest from an MS sample set by extracting the relevant data. Chromatographic peaks, ion intensity identification, and data matrix constriction were searched in the MarkerLynx software. Then, the m/z values of candidate markers were searched in the MassTRIX using the database KEGG/HMDB/LipidMaps with isotopes of Homo sapiens reference at a maximum error of 0.05 Da. The data of sorted compounds and IDs were converted to Excel spreadsheets. Accurate mass queries were conducted in compound databases (Metlin, Human Metabolome Database, PubChem, ChemSpider), and fragmentation patterns were searched in spectral databases (MassBank, NIST2014) for structural identification of the molecular formulas. PLS-DA and OPLS-DA were performed using the SIMCA-P software (Umeå, Västerbottens län, Sweden). The VIP values summarize the overall contribution and importance of each X-variable. Variables with a VIP value over 1 were considered as significant metabolites. The PLS-DA data were further analyzed via Student’s *t*-test analysis using the MedCalc software (MedCalc, Ostend, Belgium). *p*-value adjustments for multiple metabolite comparisons were conducted using Benjamini and Hochberg false discovery rate adjustment. Cross validation was performed using the MetaboAnalyst 3.0 (McGill University, Montréal, QC, Canada). For cross validation, the total data should be divided into a training set, a validation set, and a test set. Accordingly, the samples were divided into six groups, and two classes of individuals were ensured in validation sets or test sets [32]. The m/z values of the metabolites were inputted to the web-based tool MassTRIX as a reference at a maximum error of 0.05 Da.

The levels of polyamines are expressed as means ± standard deviations. Differences between two groups were confirmed by t-test using the MedCalc software (MedCalc, Ostend, Belgium). The threshold of significance was set at *p* < 0.05.

## 5. Conclusions

In conclusion, our study provides insights into the comprehensive metabolic alterations in pre- and post-operative patients with CRC. This is first study to use a metabolomics approach to examine samples form patient with CRC pre- and post-surgery. Our study confirmed differences in the overall metabolic profiles before and after surgery. In addition, we identified certain candidate markers of CRC. Specifically, the metabolic profiles of sphingolipid, steroid, and arginine metabolism were altered before and after surgery. Although metabolic profiles have been described individually in the context of CRC and other cancers, no studies had previously quantified and confirmed specific metabolites. After the untargeted metabolomics approach, we performed targeted profiling of polyamines, known cancer biomarkers. In our results, the level of PUT was significantly decreased after surgery. We suggest that these results have relevance in surgical operations because we employed an untargeted approach and targeted polyamine profiling. This method may help predict prognosis following surgery and can be readily applied to pre-surgical metabolic testing. In the future, quantitative analysis of various metabolites that are part of other metabolic pathways in cancer patients after surgery can be performed.

## Figures and Tables

**Table 1:** Differentially regulated metabolites between samples from patients with pre- and post-operative colorectal cancer.

Metabolites	Related Pathways	VIP	*p*-Value	Fold Change	Correlation Coefficient	Regulation
Gamma-aminobutyric acid	Arginine and proline metabolism	1.0023	<0.001	0.145828341	4.117358559	Up
l-Proline	Arginine and proline metabolism	1.71621	<0.001	0.248728569	3.038287404	Up
l-Threonine	Glycine, serine, and threonine metabolism	4.84114	<0.001	−0.053349474	6.563445768	Up
N_4_-Acetylaminobutanal	Lysine degradation	1.62713	<0.001	0.261500031	2.625861377	Up
Unknown (m/z = 131.0643)	NA	1.06877	<0.001	−0.080578542	6.860433801	Up
Thymine	pyrimidine metabolism	1.50327	<0.001	−0.37427256	1.606954027	Up
Mevalonic acid	Terpenoid backbone biosynthesis	1.01718	<0.001	−0.093645679	6.286436005	Up
Xanthine	Purine metabolism	1.00114	<0.001	0.516947844	2.518903854	Up
Unknown (m/z = 307.0758)	NA	1.42141	<0.001	0.423472971	7.681495389	Up
Testosterone	Steroid hormone biosynthesis	1.0227	<0.001	−0.181019259	0.467837133	Down
Phytosphingosine	Sphingolipid metabolism	1.02506	<0.001	0.283161742	4.211946967	Up
Dihydroceramide	Sphingolipid metabolism	2.90611	<0.001	0.089880246	0.05540464	Down
Prostaglandin E_2_	Arachidonic acid metabolism	1.44895	0.001	−0.052474587	46.02889156	Up
11β,21-Dihydroxy-5β-pregnane-3,20-dione	Steroid hormone biosynthesis	1.02444	0.002	−0.002157481	0.726773548	Down
Chenodeoxycholic acid	Primary bile acid biosynthesis	1.01772	<0.001	−0.197870962	31.81241423	Up
Phosphoribosyl pyrophosphate	pyrimidine metabolism	1.05047	<0.001	0.039117952	0.084114079	Down
Episterol	Steroid biosynthesis	1.58977	<0.001	−0.01518394	65.07462767	Up
Beta-Sitosterol	Steroid biosynthesis	1.34755	<0.001	0.548996098	2.874787603	Up
4α-Carboxy-4β-methyl-5α-cholesta-8,24-dien-3β-ol	Steroid biosynthesis	1.08986	<0.001	0.317512451	0.329377471	Down
Leukotriene D_4_	Arachidonic acid metabolism	2.41915	<0.001	−0.017846652	85.53840187	Up
Ceramide 1-phosphate	Sphingolipid metabolism	1.03921	<0.001	−0.167097911	0.016887882	Down
Androsterone glucuronide	Steroid hormone biosynthesis	1.023	<0.001	−0.164779789	0.022209325	Down

**Table 2:** Concentrations of nine polyamines in serum samples from pre- and post-operative patients with colorectal cancer (ng/mL).

Compound	Pre-Operative Patients (*n* = 68)		Post-Operative Patients (*n* = 68)		*p*-Value
	Mean ± SD	Median, Range	Mean ± SD	Median, Range	
N-PUT	157.49 ± 82.84	137.38, 34.62–368.14	154.86 ± 76.96	131.01, 43.88–346.82	0.858
N-CAD	28.41 ± 12.97	26.38, 8.88–82.7	28.47 ± 15.27	23.62, 9.02–63.32	0.981
DAP	14.62 ± 10	12.24, 2.53–47.05	15 ± 12.16	11.21, 2.55–46.28	0.858
PUT	28.45 ± 16.4	30.22, 2.29–63.93	18.18 ± 11.67	15.63, 4.76–80.91	4.97^−05^
CAD	245.37 ± 304.23	28.21, 11.4–803.81	210.92 ± 193.49	200.65, 5.94–665.98	0.482
N-SPD	58.08 ± 20.65	53.61, 27.38–137.7	64.93 ± 18.44	67.43, 19.22–97.34	0.166
SPD	111.11 ± 78.11	98.5, 4.61–457.76	95.72 ± 78.62	64.23, 13.44–326.68	0.274
N-SPM	139.7 ± 98.81	120.77, 13.87–426.94	109.82 ± 83.62	72.08, 18.75–353.23	0.107
SPM	1040.28 ± 1040.5	669.95, 10.89–3764.49	849.93 ± 843.35	709.89, 9.8–3683.69	0.262

N-PUT, N-acetyl putrescine; N-CAD, N-acetyl cadaverine; DAP, 1,3-diaminopropane; PUT, putrescine; CAD, cadaverine; SD, standard deviation; N-SPD, N-acetyl spermidine; SPD, spermidine; N-SPM, N-acetyl spermine; SPM, spermine.

**Table 3:** Clinical characteristics of study groups.

Characteristic	Value
**Patients (n)**	68
**Sex**	
Male	49
Female	19
**Age**	
Mean ± SD	57.52 ± 9.71
Range	20–71
**Nationality, n (%)**	Korean (100)
**Body mass index**	23.2
**Stage**	
0	5
Ι	21
II	21
III	1
IIIA	5
IIIB	9
IIIC	2
n/a	4
**Method of operation**	
Laparoscopy	55
Da Vinci	6
Low anterior	4
Left hemicolectomy	1
Right hemicolectomy	2
**Other chronic diseases, n (%)**	
None	54
Hypertension	10
Diabetes	3
Crohn’s disease	1
